# Highly immunosuppressive myeloid cells correlate with early relapse after allogeneic stem cell transplantation

**DOI:** 10.1186/s40164-024-00516-4

**Published:** 2024-05-11

**Authors:** Anne-Béatrice Notarantonio, Allan Bertrand, Romain Piucco, Ghislain Fievet, Hervé Sartelet, Laura Boulangé, Natalia de Isla, Marcelo De Carvalho Bittencourt, Sébastien Hergalant, Marie-Thérèse Rubio, Maud D’Aveni

**Affiliations:** 1 UMR 7365 CNRS, IMoPA, Université de Lorraine, 54000 Nancy, France; 2 Hematology Department, CHRU Nancy, Université de Lorraine, 54000 Nancy, France; 3 Inserm UMR_S 1256 NGERE, Université de Lorraine, 54500 Vandœuvre-les-Nancy, France; 4 Anatomopathology Department, CHRU Nancy, Université de Lorraine, 54000 Nancy, France; 5 Immunology Laboratory, CHRU Nancy, Université de Lorraine, 54000 Nancy, France

**Keywords:** Allogeneic stem cell transplantation, Relapse, MDSC

## Abstract

**Background:**

Allogeneic hematopoietic stem cell transplantation (allo-HSCT) is the only curative treatment for myeloid malignancies such as some acute myeloid leukemias (AML) and high-risk myelodysplastic syndromes (MDS). It aims to eradicate the malignant clone using immunocompetent donor cells (graft-versus-leukemia effect, GVL). Unfortunately, relapse is the primary cause of transplant failure mainly related on HLA loss or downregulation and upregulation of inhibitory ligands on blasts which result in donor immune effector dysfunctions.

**Methods:**

Between 2018 and 2021, we conducted a monocentric prospective study including 61 consecutive patients transplanted for AML or high-risk MDS. We longitudinally investigated immune cells at days + 30, + 90 and + 180 post-transplant from bone marrow and peripheral blood. We assessed the dynamics between myeloid derived suppressor cells (MDSCs) and T-cells.

**Results:**

Among the 61 patients, 45 did not relapse over the first 12 months while 16 relapsed during the first year post-transplant. Through months 1 to 6, comparison with healthy donors revealed an heterogenous increase in MDSC frequency. In all recipients, the predominant MDSC subset was granulocytic with no specific phenotypic relapse signature. However, in relapsed patients, in vitro and in vivo functional analyses revealed that MDSCs from peripheral blood were highly immunosuppressive from day + 30 onwards, with an activated NLRP3 inflammasome signature. Only circulating immunosuppressive MDSCs were statistically correlated to circulating double-positive Tim3+LAG3+ exhausted T cells.

**Conclusion:**

Our simple in vitro functional assay defining MDSC immunosuppressive properties might serve as an early biomarker of relapse and raise the question of new preventive treatments targeting MDSCs in the future.

*Trial registration* NCT03357172

**Supplementary Information:**

The online version contains supplementary material available at 10.1186/s40164-024-00516-4.

Allogeneic hematopoietic stem cell transplantation (allo-HSCT) is the only curative treatment for myeloid malignancies such as high-risk acute myeloid leukemias (AML) and myelodysplastic syndromes (MDS). Allo-HSCT holds promise for long-term disease control. Donor alloreactive T cells can both eliminate residual tumor cells (graft-versus-leukemia, GVL) and damage normal host tissues, causing the graft-versus-host disease (GVHD). Currently, relapse remains the main cause of death after allo-HSCT and is attributed to the loss of the GVL effect based on diverse mechanisms such as genomic HLA loss, transcriptional changes of HLA class II expression and upregulation of inhibitory ligands on blasts [1]. Myeloid derived suppressor cells (MDSCs) represent a heterogeneous cell population and their role in the tumor escape from the allogeneic immune response remains a subject of debate [2]. In mouse models, MDSCs isolated from tumor-bearing mice [3], G-CSF treated mice [4] or in vitro cultures [3, 5, 6], infused on day + 0 or post-transplant, alleviate GVHD while preserving GVL effect. In human cohorts, higher frequencies of M-MDSC were described as early parameters strongly associated to subsequent relapse in recipients [7, 8] with conflicting results [9].

In the present study, we investigated MDSCs and early T-cell differentiation in 61 patients allografted for AML or high-risk MDS during the first 6 months posttransplantation to identify a specific immune signature of relapse. Patient characteristics and outcomes are described in Additional file 1: Tables S1 and S2, according to 2 groups: the “R” group defined by early relapse occurring within 12 months following allo-HSCT and the “NR” group defined by no relapse over the first 12 months after allo-HSCT.

MDSCs were similarly observed in all recipients and were predominantly represented by the granulocytic subset (Fig. 1A). As previously described [10], in comparison with healthy donors, the percentages and numbers of MDSCs among circulating CD45+ leukocytes were heterogeneously increased within 6 months after allo-HSCT (Fig. 1B). Concomitantly, we did not identify a specific pattern of peripheral blood T-cell differentiation in patients with early relapse (Fig. 1C). As previously described [9], T-cell differentiation was largely skewed toward effector memory T cells (T_EM_ and T_EMRA_) (Fig. 1D).

**Fig. 1:**
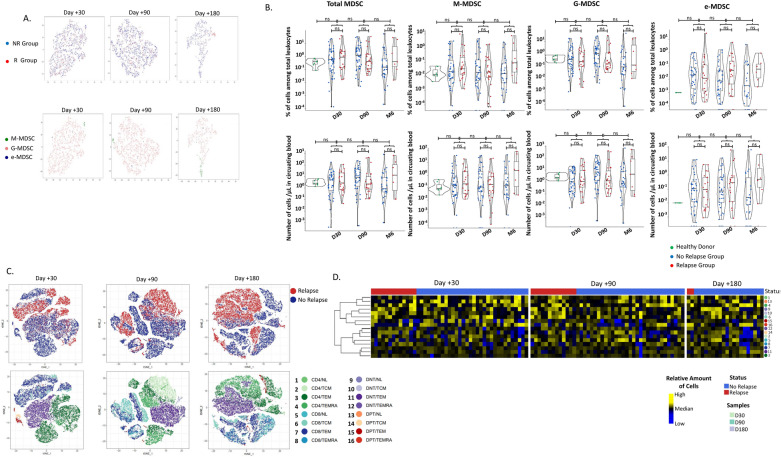
MDSC and T-cell landscape during the first 6 months after allo-HSCT. **A** Unsupervised analyses of MDSC circulating in recipient peripheral blood at day + 30, day + 90 and day + 180. Circulating MDSC are similarly observed in NR and R groups. Granulocytic MDSC are the main subset among the MDSC. **B** Percentage and absolute numbers of MDSC subpopulations values across patients in “R” group and patients in “NR group”, compared to healthy donor. The bar indicates median value, the box represents the lower and upper quartile, the violin plots represents the density. No significant statistical difference was observed at D30, D90 and D 180 (M6), between the three groups. P-values correspond to Wilcoxon signed-rank tests. All tests are NS = not significant. For total MDSC, we observe 0.40% ± 0.08% in healthy donors; 3.70% ± 1.92%, 3.36% ± 1.61% and 7.11% ± 4.36% in “R” group and 2.44% ± 0.71%, 4.11% ± 1.12% and 2.52% ± 1.74% in NR group at D30, D90 and M6 respectively. For total MDSC, we observe 3.44 ± 0.73 MDSC/µL in circulating blood in healthy donors; 14.71 ± 5.66, 47.33 ± 25.70 and 134.1 ± 94.02/µL in “R” group at D30, D90 and M6, and 12.42 ± 4.4, 29.97 ± 8.05 and 36.02 ± 28.59 in “NR” group. **C** t-Distributed Stochastic Neighbor Embedding (tSNE) analyses of T-cells circulating in recipient peripheral blood at Day + 30, Day + 90 and Day + 180. The different T-cell subsets are similarly observed in NR (blue) and R (red) groups. Memory T cells are the main subset among T-cell subsets. In each group, 5000 cells from 5 randomly selected patients were pooled together then reduced to tSNE representation. **D** Hierarchical clustering heat map of T cells circulating in recipient peripheral blood. PBMCs from 61 AML/MDS patients with (n = 16) and without (n = 45) documented subsequent tumor relapse were collected at Day + 30, Day + 90 and Day + 180 after transplantation and analyzed by flow cytometry according to their differentiation profile. No specific T-cell subpopulations were associated with relapse

Initial experiments exploring MDSC immunosuppressive properties were performed using a CD3/CD28 microbead-based assay. We observed significant inhibition of T-cell proliferation by MDSCs (Fig. 2A). However, visual examination suggested MDSC sequestration of CD3/CD28 microbeads, as previously demonstrated with murine MDSCs [11]. We therefore consistently evaluated MDSC immunosuppressive properties in a plate-bound anti-CD3/28 T-cell stimulation assay. We did not observe T-cell proliferation suppression in the presence of MDSCs in the NR group at 1, 3 and 6 months after allo-HSCT. Conversely, MDSCs sorted from the R group exhibited an immunosuppressive effect since day + 30 (Fig. 2B) with an activated NLRP3 inflammasome (Fig. 2C). Only immunosuppressive MDSC confirmed in vitro, protect NSG mice from GVHD (Fig. 2D), with a lower GVHD histopathological score (Fig. 2F). Finally, we found strong positive correlations between immunosuppressive M-MDSCs and exhausted CD8^+^ T cells circulating in the R group but no correlation with Tregs (Fig. 2E).

**Fig. 2:**
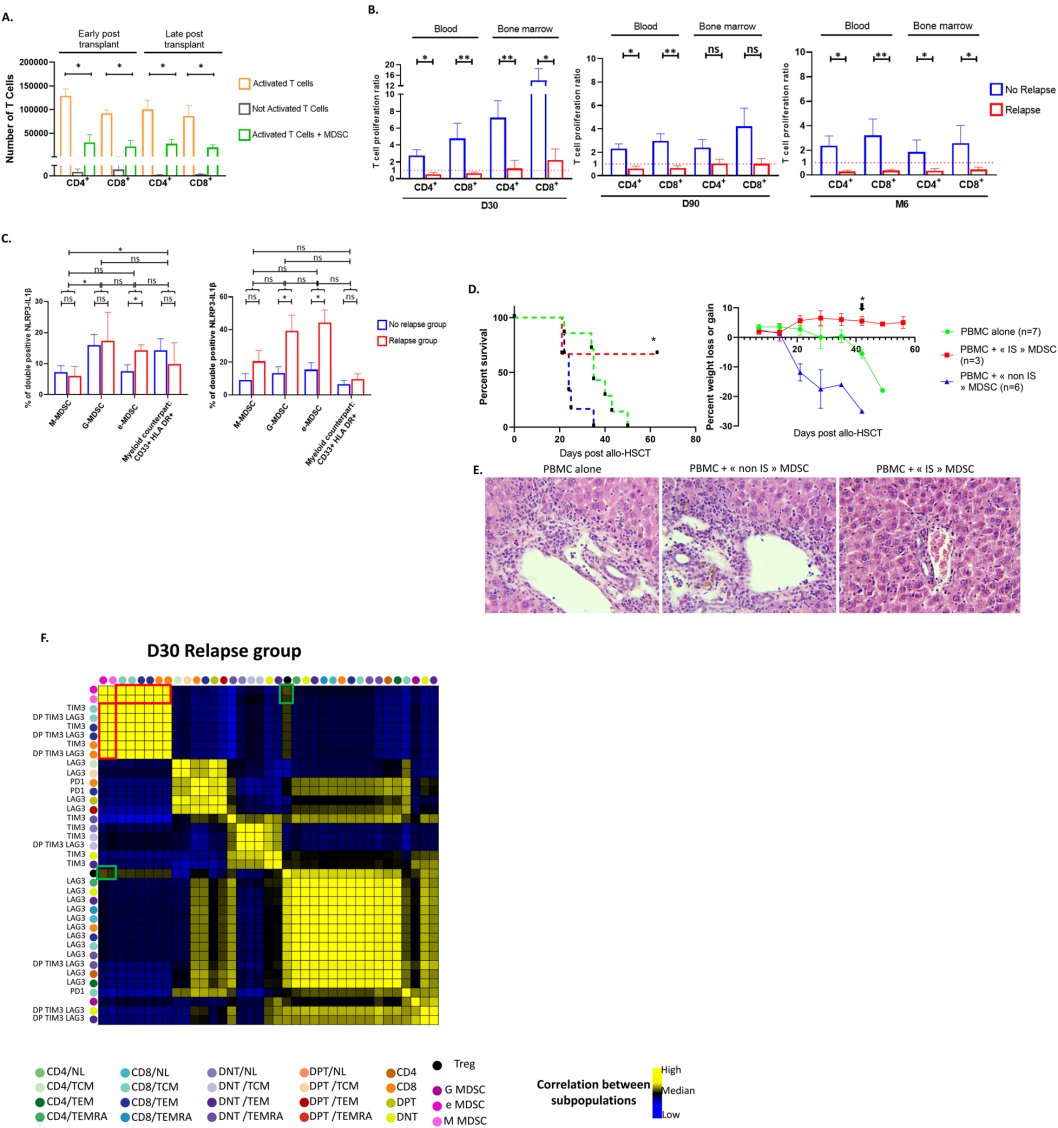
Immunosuppressive MDSCs circulating on day + 30 are observed in early relapse and correlate with exhausted T cells. **A** CD3/CD28 microbead-based assay. For early post-transplant (samples before day + 90), T-cells were activated and then cultured alone, or with MDSC cells at a 1:1 ratio (50,000 T cells:50,000 MDSCs) sorted from circulating blood of recipients before days 60 post-transplant (n = 4). For late post-transplant, T-cells were activated and then cultured alone, or with MDSC cells at a 1:1 ratio (50,000 T cells:50,000 MDSCs) sorted from circulating blood of recipients after day 90 post-transplant (n = 4). For early post-transplant, mean number of proliferating CD4+ and CD8+ T-cells activated alone (orange) were respectively 129,172 ± 14,559 and 92,065 ± 6 945, versus 30,812 ± 16,727 and 22,049 ± 12,815 in co-culture with MDSCs (green) (*p = 0.0286). For late post-transplant (samples after day + 90), mean number of proliferating CD4+ and CD8+ cells activated alone were 100,255 ± 19,412 and 86,805 ± 21,707 versus 28,138 ± 8601 and 19,943 ± 5585 in culture with MDSCs (*p = 0.0286). **B** Plate-bound anti-CD3, anti-CD28 assay. The ratio corresponds to the calculation of the number of proliferating T-cells activated in co-culture with MDSCs divided by the number of proliferating T-cells activated alone in culture. Ratio was calculated for co-cultures performed with MDSCs sorted from recipient blood or bone marrow at day 30 (D30), day 90 (D90) and 6 months (M6), with a 1 MDSC:1T-cell ratio. Immunosuppressive MDSC are defined when a ratio of < 1 is observed in the MDSC/T-cell coculture. At D30, ratio for proliferating CD4+ T cells cultured with MDSCs from recipient blood are 2.74 ± 0.70 in NR group (n = 13) versus 0.53 ± 0.21 in R group (n = 3) (*p = 0.0143) and 4.76 ± 1.81 vs 0.67 ± 0.12 for CD8+ (**p = 0.0036). At D30, ratio for proliferating CD4+ T cells cultured with MDSCs from recipient bone marrow are 7.24 ± 2.00 in NR group (n = 10) versus 1.22 ± 0.96 in R group (n = 5) (**p = 0.0080) and 14.09 ± 4.48 vs 2.21 ± 1.32 for CD8+ (*p = 0.0400). At D90, ratio for proliferating CD4+ T cells cultured with MDSCs from recipient blood are 2.31 ± 0.39 in NR group (n = 15) versus 0.59 ± 0.22 in R group (n = 3) (*p = 0.0172) and 2.95 ± 0.62 vs 0.66 ± 0.19 for CD8+ (**p = 0.0098). At D90, ratio for proliferating CD4+ T cells cultured with MDSCs from recipient bone marrow are 2.41 ± 0.66 in NR group (n = 9) versus 1.036 ± 0.38 in R group (n = 5) (p = 0.1194) and 4.21 ± 1.55 vs 1.024 ± 0.44 for CD8+ (p = 0.1194). At M6, ratio for proliferating CD4+ T cells cultured with MDSCs from recipient blood are 2.38 ± 0.79 in NR group (n = 16) versus 0.27 ± 0.09 for relapse group (n = 3) (*p = 0.0144) and 3.32 ± 1.32 vs 0.35 ± 0.08 for CD8+ (**p = 0.0041). At M6, ratio for CD4 + cultured with MDSCs from recipient bone marrow are 1.87 ± 0.97 in NR group (n = 9) versus 0.33 ± 0.17 in R group (n = 5) (*p = 0.0290) and 2.59 ± 1.43 vs 0.43 ± 0.20 for CD8+ (*p = 0.0120). **C** Percentage of myeloid cells with activated inflammasome. We evaluated the % of double positive (NLRP3+ and IL1β+), in three MDSC subsets (M-MDSC, G-MDSC, e-MDSC) and the myeloid counterpart (CD33+HLA-DR+) in peripheral blood and in bone marrow. In peripheral blood, the percentage of double positive (NLRP3 and IL1β) in each four myeloid subset is compared in the “NR” group (n = 26) versus the “R” group (n = 4). This percentage is statistically different in the e-MDSC subset (7.55% ± 2.07% vs 14.25% ± 1.85%, respectively, *p = 0.0315). In the M-MDSC, G-MDSC and the myeloid counterpart, the percentages are comparable with 7.22% ± 2.11% vs 6.05% ± 3.03%, 15.91% ± 3.51% vs 17.42% ± 9.17% and 14.28% ± 3.75% vs 9.87% ± 6.86%, respectively. In bone marrow, the percentage of double positive (NLRP3 and IL1β) in each four myeloid subset is compared in the “NR” group (n = 15) versus “R” group (n = 4). This percentage is statistically different in G-MDSC and e-MDSC subsets (13.43% ± 3.79% vs 39.22% ± 9.58%, *p = 0.0139 and 15.47% ± 4.35% vs 44.32% ± 7.63%, *p = 0.0139, respectively). There is also a trend in M-MDSC (9.09% ± 3.87% vs 20.56% ± 6.62%, p = 0.0769). However in the CD33+HLA DR+ subset, there is no significant difference in the « NR» group vs «R» group, with 6.58% ± 2.36% vs 9.79% ± 3.05% (p = 0.2256), respectively. **D** Median survival in mice receiving PBMC with MDSC from “R” group was not reached by day 60 vs 35 days for mice injected with PBMC alone vs 24 days for mice injected with PBMC and MDSC from “NR” group (p = 0.0281). Data shown represent pooled results from 2 independent experiments. Results were compared with Kaplan–Meier survival curves. Percent weight variations were at day + 42 post-HSCT: + 4.60 ± 0.64% in mice injected with PBMC and MDSC from “R group” (n = 3) vs − 1.91 ± 2.93% in mice injected with PBMC alone (n = 7) (*p* = 0.05), vs − 11.08 ± 4.46% in mice injected with PBMC and MDSC from “NR” group (n = 6) (p = 0.0087). Data were compared using Student’s unpaired t-test. **E** Representative histological analyses of GVHD target organs in each group. Portal inflammation with diffuse or nodular infiltrate of mononuclear cells, and endothelitis observed in the group with “PBMC alone” and the group “PBMC+ non IS MDSC”. **F** Correlation heatmaps on day + 30 between MDSC and exhausted T-cell subsets in the “R” group (n = 6) highlighted in red. Color scale: blue (negative correlation); yellow (positive correlation); black (no correlation). Spearman’s tests on semi-automatic-gating cell counts after removal of unpopulated and insufficiently populated subpopulations. Exhaustion markers for each subset are displayed on the left

If MDSCs preserve the GVL effect in mouse models [3, 5], only few prospective studies reported a correlation between higher M-MDSC frequencies in patients in the 30 days following allo-HSCT and a higher probability of relapse [7, 8]. Other studies have suggested in vitro that MDSCs might suppress donor T cell proliferation and Th1 differentiation and promoted Treg development [10, 12]. The strength of our study is that we prospectively studied fresh recipient samples, and we focused on myeloid malignancies transplanted after a “Flu-Bu” regimen with HLA-matched donors, avoiding irradiation and cyclophosphamide in the preparative regimen and avoiding post-transplant G-CSF and cyclophosphamide that might induce MDSCs, as suggested in mouse models. Our study highlights for the first time that MDSCs defined only by phenotypic features should be interpreted with caution in the allo-HSCT context. The accumulation of immature myeloid cells with an MDSC-like phenotype seemed to be linked to inflammatory hematopoiesis after allo-HSCT. Of note, it has been previously demonstrated in mice that under inflammatory conditions, MDSCs accumulate and rapidly differentiate away from immature lineage cells, losing their immunosuppressive properties. Our in vitro T-cell proliferation assay seems robust, as immunosuppressive properties assessed in vitro were confirmed in vivo. Moreover, this assay can be easily performed in immunology laboratories. Our study underlined that the functional assay based on anti-CD3/CD28 microbeads for investigating MDSC immunosuppressive properties [10, 12] should be avoided, as it artificially blocks T-cell proliferation by bead phagocytosis from myeloid cells. Consequently, we conclude that both immunophenotyping and functional assays are needed to clearly identify MDSCs in the context of allo-HSCT.

In the context of allo-HSCT, we propose to distinguish: (i) the accumulation of immature myeloid cells with an MDSC-like phenotype that solely results from alloreactive inflammation and (ii) circulating immunosuppressive MDSCs that correlate with exhausted CD8+ T cells. These MDSC display a specific activated NLRP3 inflammasome signature, particularly in bone marrow, suggesting a probable cancer persisting microenvironment (with consequently an early relapse).

### Supplementary Information

**Additional file 1.** Supplementary data: Material and methods, Supplementary tables and figure.

## Data Availability

All experimental data are registered in laboratory note books and all clinical and case report forms (CRFs) are reported in electronic questionnaires.

## Abbreviations

Allo-HSCT: Allogeneic hematopoietic stem cell transplantation
AML: Acute myeloid leukemia
ATLG: Anti-T lymphocyte globulin
CMV: Cytomegalovirus
CR: Complete remission
ELN: European Leukemia Network
GVHD: Graft versus host disease
GVL: Graft versus leukemia
HLA: Human leukocyte antigen
IPSS-R: Revised international prognostic scoring system
LAG3: Lymphocyte activation gene-3
NLRP3: “NOD-like” receptor family, pyrin domain containing 3
MHC: Major histocompatibility complex
MDS: Myelodysplastic syndrome
MDSC: Myeloid derived suppressor cell
MRD: Matched related donor
MUD: Matched unrelated donor
Tim 3: T-cell immunoglobulin and mucin containing protein-3 (Tim3)
PD-1: Programmed cell death 1
PR: Partial remission
WHO: World Health Organization
