# Supplementary material for: Highly immunosuppressive myeloid cells correlate with early relapse after allogeneic stem cell transplantation
Source: Exp Hematol Oncol. 2024 May 11;13:50. doi: 10.1186/s40164-024-00516-4 (PMC11088072; doi:10.1186/s40164-024-00516-4)
Supplement: Supplementary file 1 — Additional file 1. Supplementary data: Material and methods, Supplementary tables and figure. [file 40164_2024_516_MOESM1_ESM.docx]

**SUPPLEMENTARY DATA**

**MATERIAL AND METHODS**

*Study design and data collection*

The inclusion criteria were as follows: age ≥18 years and allo-HSCT with an HLA-matched related donor (MRD) or unrelated donor (MUD). Conditioning regimens consisted of

*myeloablative conditioning: “MAC”: FluBu4, 40mg/m2/day IV fludarabine for 4 days from day-6 to day-3 and 3.2mg/kg/day IV busulfan for 4 days from day-6 to day-3) for patients aged between 18-55 years with no significant comorbidities.

*Reduced toxicity and reduced intensity conditionings: “RTC/RIC” (FluBu3: 40mg/m2/day IV fludarabine for 4 days from day-6 to day-3 and 3.2mg/kg/day IV busulfan for 3 days from day-5 to day-3; and FluBu2: 40mg/m2/day IV fludarabine for 4 days from day-6 to day-3 and 3.2mg/kg/day IV busulfan for 2 days from day-4 to day-3) was indicated if at least one of the following parameters was present: i) Patient ≥ 56 years old, ii) Heavily pre-treated patients who received and autologous hematopoietic SCT or with more than 2 lines of chemotherapy before allo-HSCT, iii) Patients with poor performance status because of significant medical comorbidities.

The hematopoietic stem cell source was peripheral blood stem cells (PBSCs) mobilized by G-CSF. For all patients, *in vivo* T-cell depletion was performed with anti-T-lymphocyte globulin (ATLG, 30 mg/kg for MUD and 15 mg/kg for MRD transplants). GVHD prophylaxis included administration of cyclosporin A starting on day -1 associated with mycophenolate mofetil starting on day +1 with RTC/RIC; cyclosporin A and a short course of methotrexate (15 mg/m^2^ on day +1 and 10 mg/m^2^ on days +3 and +6) with MAC. MDS diagnosis was defined according to the 2016 World Health Organization (WHO) classification; high-risk MDS was defined by the classical International Prognostic Scoring System (IPSS)[1] at diagnosis (≥1.5) regardless of the revised IPSS (R-IPSS)[2]; only AML patients with criteria for allo-HSCT according to ELN 2017 were included [3].

The exclusion criteria were patients who received allo-HSCT from an alternative donor (HLA-mismatched donor, haploidentical donor, or umbilical cord blood). Patients with secondary AML/MDS in the 5 years following the treatment of a solid tumor and patients under treatment for a serious infection before transplantation (including mycobacterial infection, aspergillosis or mucormycosis).

*Patient cohort*

Between 2018 and 2021, we considered 61 leukemic and MDS patients who underwent a first allogeneic hematopoietic stem cell transplantation. A total of 61 patients with high-risk MDS (n=15) and AML (n=46) were included in this study. Detailed characteristics of the patients are summarized in the **supplementary table**. Peripheral blood samples (10 mL in an ethylenediamine tetraacetic acid -EDTA- tube) and bone marrow samples (3 mL in an EDTA tube) from recipients were collected at days +30, +90, and +180 after transplantation **(Figure S1A)**.

*Chimerism*

Quantitative analysis of chimerism after allo-HSCT using fluorescent microsatellite PCR [4] was routinely performed on whole bone marrow cells or blood cells.

*Flow cytometry*

Peripheral blood and bone marrow cells from recipients were stained with appropriately labeled monoclonal antibodies (mAbs) against CD34 (Clone 651, Sony), CD11b (Clone LM2/1, Invitrogen), CD33 (Clone WM-53, Invitrogen), CD14 (Clone M5E2, BD Biosciences), CD15 (Clone H198, Invitrogen), HLA-DR (Clone I243, Sony), PD-L1 (Clone 29E2A3, Sony) and lineage markers CD3 (Clone HIT3a, Sony), CD19 (Clone 4G7, Sony) for the MDSC panel and against CD3 (Clone UCHT1, BD Biosciences), CD19 (Clone S/25C1, Sony), CD4 (Clone SK3, BD Biosciences), CD8 (Clone SK1, BD Biosciences), CD197 (Clone G043H7, Sony), CD95 (Clone DX2 BD Biosciences), CD45RA (Clone 5H9, BD Biosciences), LAG-3 (Clone 7H2C65, Sony), TIM-3 (Clone F38-2E2, Sony), and PD-1 (Clone EH12.2H7, Sony), CD127 (Clone HIL-7R-M2, BD Biosciences), CD25 (Clone 2A3, BD Biosciences), for the T-cell panel. Samples were analyzed on a Gallios cytometer (BD Biosciences), and cell events were further processed using Kaluza 2.1 software.

Among the PBMCs, we analyzed MDSCs by focusing on lineage and HLA-DR-negative cells and then gating on double-positive CD33^+^ CD11b^+^ cells in supervised and unsupervised strategies (**Figures S1B and S1C**). MDSCs are defined by flow cytometry as Lin− HLA-DR low CD33high CD11b+ cells. We observed that a very small subset of myeloid cells corresponded to the MDSC phenotype definition. Monocytic MDSCs (M-MDSCs) are CD14+ CD15+/-, granulocytic MDSCs (G-MDSCs) are CD15+ but CD14−, and early-stage or promyelocytic MDSCs (called e- or P-MDSCs) are CD14− CD15−. We further investigated T-cell subset frequencies (TN, naïve T cells CD3+ CD45RA+ CCR7+/TCM, central memory T cells CD3+ CD45RA- CCR7+/TEM, effector memory T cells CD3+ CD45RA- CCR7-/TEMRA, terminal effector T cells CD3+ CD45RA+ CCR7-) on CD4+, CD8+, double-positive (DP) CD4+ CD8+ and double-negative (DN) CD4- and CD8- T cells. (**Figure S2**). Hierarchical clustering of the T-cell compartment was performed on relative abundances for each gated subpopulation. We named the subpopulations as follows:

1= CD4+ naïve T cells (T_N_, CD3+CD4+CD45RA+CCR7+),

2= CD4+ central memory T cells (T_CM_, CD3+CD4+CD45RA- CCR7+),

3= CD4+ effector memory (T_EM_, CD3+ CD4+ CD45RA-CCR7-),

4= CD4+ terminal effectors (T_EMRA_ CD3+ CD4+ CD45RA+ CCR7-),

5= CD8+ naïve T cells (T_N_, CD3+CD8+CD45RA+CCR7+),

6= CD8+ central memory T cells (T_CM_, CD3+CD8+CD45RA- CCR7+),

7= CD8+ effector memory (T_EM_, CD3+ CD8+ CD45RA-CCR7-),

8= CD8+ terminal effectors (T_EMRA_ CD3+ CD8+ CD45RA+ CCR7-),

9= CD4-CD8- naïve T cells (T_N_, CD3+CD4-CD8-CD45RA+CCR7+),

10= CD4-CD8- central memory T cells (T_CM_, CD3+CD4-CD8-CD45RA- CCR7+),

11= CD4-CD8- effector memory (T_EM_ CD3+ CD4-CD8-CD45RA-CCR7-),

12= CD4-CD8- terminal effectors (T_EMRA_ CD3+ CD4-CD8-CD45RA+ CCR7-),

13= CD4+CD8+ naïve T cells (T_N_, CD3+CD4+CD8+CD45RA+CCR7+)

14= CD4+CD8+ central memory T cells (T_CM_, CD3+CD4+CD8+CD45RA- CCR7+),

15= CD4+CD8+ effector memory (T_EM_ CD3+ CD4+CD8+CD8-CD45RA-CCR7-),

16= CD4+CD8+ terminal effectors (T_EMRA_ CD3+ CD4+CD8+CD45RA+ CCR7-).

*MDSC isolation and functional assays*

MDSCs were purified from the Ficoll-purified peripheral blood mononuclear cell (PBMC) fraction by negative selection using an HLA-DR depletion kit (Miltenyi Biotec, Bergisch Gladbach, Germany) and thereafter a CD33-positive selection kit (Miltenyi Biotec) as previously described [5]. Functional analyses *in vitro* and *in vivo* were performed as previously described [6].

*Proliferation assays:* T cells were purified by negative selection using a pan T-cell depletion kit (Miltenyi Biotec). Purity was routinely above 98% (not shown). T-cell activation (5 x 10^4^ cells/well) was performed by nonspecific TCR activation in a 96-well culture plate coated with 10 µg/mL anti-CD3 mAb (Biotechne) and 10 µg/mL anti-CD28 mAb (Biotechne) for 2 hours before the addition of T cells. MDSCs were plated at 5 x 10^4^/well (1:1 ratio). Cells were incubated in custom RPMI 1640 with 10% fetal calf serum and 100 U/mL penicillin‒streptomycin (Life Technologies). T cells were stained with 5 μM carboxyfluorescein succinimidyl ester (CFSE) (Invitrogen). T-cell proliferation was analyzed after 5 days of coculture and expressed as the ratio of the number of activated T cells obtained in the presence of MDSCs to the number of T cells activated without MDSCs. Thus, an immunosuppressive assay was characterized by a ratio under the threshold value of 1 **(figures S1D)**.

*-Mice:* NOD.Cg-PrkdcscidIl2rgtm1WjI/SzJ (NSG) mice were purchased from Charles River (L’Arbresle, France). All mice were bred and housed in a specific pathogen-free facility in microisolator cages and used at 8 to 12 weeks of age according to protocols approved by the local ethics committee (Referral n°26194). NSG mice were irradiated using 2 Gy total body irradiation by X-ray on day -1 followed by intravenous infusion in the caudal vein of 5x10^6^ human PBMCs. MDSCs were sorted from patient peripheral blood between day +45 and +60 posttransplantation when the T-cell proliferation assay on day 30 was performed and distinguished the “IS” group (immunosuppressive MDSCs, as the T-cell proliferation ratio was <1) *versus* the “non-IS” group (non-immunosuppressive MDSCs, as the T-cell proliferation ratio was >1). The control group was transplanted with only 5x10^6^ human PBMCs per mouse on day 0, the other groups were co-transplanted with 1x10^6^ MDSCs per mouse on day 0. For all recipient mice, PBMCs were harvested from the same donor. Mice were monitored for survival and weight twice a week. Tissues from GVHD target organs (liver, intestine and skin) were embedded in paraffin, sectioned, and stained with hematoxylin, eosin and safran. Photographs were taken using 200x or 400x magnification with an optical Carl-Zeiss AXIO Imager A1m microscope (Carl-Zeiss). Blinded histopathological assessments of GVHD were performed. In particular, liver damage was scored according to the severity of portal infiltrate, biliary damage, centrilobular vein endotheliitis and apoptosis. Histopathological analysis was performed as previously described [7]: 0, absent; 1, minimal; 2, mild and diffuse; 3, moderate; and 4, severe.

*Clinical statistical analyses*

Patient and disease characteristics and Disease Risk Index [8] were reported using descriptive statistics. Categorical variables are described as percentages, while continuous variables are reported as the mean ± standard deviation (SD) or the median interquartile range (IQR), depending on their distribution. The comparative analysis of patient characteristics before transplantation between conditioning regimens was performed using the Chi2 test or Fisher’s exact test for categorical variables. Student’s t test or the Wilcoxon test was used for continuous variables. For these variables, we further checked for composition biases between the “R” and “NR” group with a generalized linear model and observed no confounding factors is a multivariable setup (data not shown). Relapse was defined by standard hematological criteria. Acute [9] and chronic GVHD [10] were diagnosed and graded using established criteria. Cox proportional hazard models were used to perform univariable (relapse status) and multivariable survival analyses (accounting for DRI, age, CMV, conditioning and donor statuses). Models’ validity were tested with logrank and Wald tests (both p-values < 1e-07) and the proportional hazard hypothesis was checked beforehand (all variable and covariable p-values > 0.05). Survival analyses were performed under R with packages *survival* and *survminer* .

*Downstream flow cytometry data analyses*

Cell events were acquired on the Gallios Cytometer. Nonparametric tests, such as the Mann‒Whitney, Wilcoxon and log-rank tests, were performed with GraphPad Prism 8 software). Additional flow cytometry statistics were performed using FlowJo (v10.6) and the Cytobank platform (Beckman Coulter). Other statistical and main bioinformatics analyses were performed under R (v4.1 and later).

After cell event acquisition, flow cytometry data were first gated manually with FlowJo as described above. They were also processed in an unsupervised manner according to the following bioinformatics pipeline: 1) Data were compensated according to the parameters indicated by the cytometer and then subjected to stringent quality control. 2) First, they were time gated. Biased, disrupted and outlier flows corresponding to entire segments of time were discarded. This step was visually controlled to ensure that the resulting flows were linear and interpretable. Second, FlowClean (v1.36) [11] was used to flag and report events with other acquisition issues. 3) The remaining cell events were normalized channel-by-channel and whole-cohort-wise using GaussNorm under flowStats (v4.10) [12], leading to multiple alignments of high-density regions in each channel. The min peaks parameter was adjusted according to the number of density peaks for each channel. 4) Dead cells and singlets were removed with appropriate preliminary gating. 5) Depending on the dataset (MDSCs or T cells), semiautomatic gating strategies were developed from multiple tools available in the R packages openCyto (v2.10.1)[13]and flowWorkspace (v4.10.1, via Bioconductor) [14]. Briefly, gating was mainly based on successions of rectangular gates defined automatically by best-fit parameters. After visual inspection, each gating was finely tuned and manually corrected if needed. 6) Pregated T-cell (CD3+) and MDSC (HLA-DR-_LIN-) subpopulations were then analyzed using unsupervised algorithms such as t-SNE (v0.16) and hierarchical clustering, embedding comparisons of marker expression between biological groups. We aimed to classify samples according to their relative percentage for each T-cell subpopulation.

Clustering was performed with median-centered data and Pearson’s correlation distance as dissimilarity metrics. Heatmaps were rendered with ComplexHeatmap (v2.14) and main graphics under ggplot2 (v3.4). All R (v4.2.2) packages were acquired and used via BioConductor (v3.16). Correlation heatmaps were rendered with corplot (v0.92) on gated data counts.

**REFERENCES**

1. Greenberg P, Cox C, LeBeau MM, et al. International scoring system for evaluating prognosis in myelodysplastic syndromes. Blood 1997;89:2079-2088.

2. Greenberg PL, Tuechler H, Schanz J, et al. Revised international prognostic scoring system for myelodysplastic syndromes. Blood 2012;120:2454-2465.

3. Dohner H, Estey E, Grimwade D, et al. Diagnosis and management of AML in adults: 2017 ELN recommendations from an international expert panel. Blood 2017;129:424-447.

4. Hancock JP, Goulden NJ, Oakhill A, et al. Quantitative analysis of chimerism after allogeneic bone marrow transplantation using immunomagnetic selection and fluorescent microsatellite PCR. Leukemia 2003;17:247-251.

5. Vendramin A, Gimondi S, Bermema A, et al. Graft monocytic myeloid-derived suppressor cell content predicts the risk of acute graft-versus-host disease after allogeneic transplantation of granulocyte colony-stimulating factor-mobilized peripheral blood stem cells. Biol Blood Marrow Transplant 2014;20:2049-2055.

6. D'Aveni M, Rossignol J, Coman T, et al. G-CSF mobilizes CD34+ regulatory monocytes that inhibit graft-versus-host disease. Sci Transl Med 2015;7:281ra242.

7. Gao Y, Shan W, Gu T, et al. Daratumumab Prevents Experimental Xenogeneic Graft-Versus-Host Disease by Skewing Proportions of T Cell Functional Subsets and Inhibiting T Cell Activation and Migration. Front Immunol 2021;12:785774.

8. Armand P, Kim HT, Logan BR, et al. Validation and refinement of the Disease Risk Index for allogeneic stem cell transplantation. Blood 2014;123:3664-3671.

9. Harris AC, Young R, Devine S, et al. International, Multicenter Standardization of Acute Graft-versus-Host Disease Clinical Data Collection: A Report from the Mount Sinai Acute GVHD International Consortium. Biol Blood Marrow Transplant 2016;22:4-10.

10. Jagasia MH, Greinix HT, Arora M, et al. National Institutes of Health Consensus Development Project on Criteria for Clinical Trials in Chronic Graft-versus-Host Disease: I. The 2014 Diagnosis and Staging Working Group report. Biol Blood Marrow Transplant 2015;21:389-401 e381.

11. Fletez-Brant K, Spidlen J, Brinkman RR, et al. flowClean: Automated identification and removal of fluorescence anomalies in flow cytometry data. Cytometry A 2016;89:461-471.

12. Hahne F, Khodabakhshi AH, Bashashati A, et al. Per-channel basis normalization methods for flow cytometry data. Cytometry A 2010;77:121-131.

13. Finak G, Frelinger J, Jiang W, et al. OpenCyto: an open source infrastructure for scalable, robust, reproducible, and automated, end-to-end flow cytometry data analysis. PLoS Comput Biol 2014;10:e1003806.

14. Pezzotti N, Lelieveldt BPF, Van Der Maaten L, et al. Approximated and User Steerable tSNE for Progressive Visual Analytics. IEEE Trans Vis Comput Graph 2017;23:1739-1752.

Legend to the supplementary tables

Table 1. Patient characteristics.

Table 2. Patient outcomes.

Legends to the supplementary figure

Supplementary Figure 1. Longitudinal analyses of bone marrow and peripheral blood after allogeneic stem cell transplantation.

A. Sixty-one patients with AML/MDS were included in the longitudinal cohort. Blood and bone marrow were collected on day +30, day +90 and day +180 after transplantation. PBMCs were analyzed by flow cytometry, and if enough cells were collected, functional assays were performed.

B. Overall survival. Description of the overall survival in the “R” group (in red) and in the “NR” group in blue. In multivariate analysis, only the DRI is correlated to a poorer survival (p=0.01).

C. Comparable gating strategies for MDSCs with supervised and unsupervised methods.

D. Analysis of T cell proliferation by flow cytometry. On this example, we observe 162 615 with 95.19% of proliferating CD4+T cells, and 190 511 with 98.04% of proliferating CD8+ T cells when T cells are activatd alone. In coculture with MDSCs, we observe 82 404 with 97.51% of proliferating CD4+T-cells and 101 663 with 96.82% of proliferating CD8+ T cells. Of note, the reduction in the number of proliferating T cells was not associated with reduced proportions of proliferating T cells, which were similar in T cells activated alone and T cells cocultured with MDSCs from either the NR or R group.

Supplementary Tables. Table 1. Patient characteristics

| **Characteristic** | NR (n=45) | | R (n=16) | |  |
| --- | --- | --- | --- | --- | --- |
| **Median recipient age at transplantation, years (range)** | 60 | (23-71) | 58 | (19-74) | P=0.45 |
| **Median follow-up, days (range)** | 1313 | (41-2132) | 271 | (53-1177) | **P <0.0001** |
| **Disease Risk Index (DRI)** |  |  |  |  | **P=0.06** |
| Very high | 0 | (0) | 1 | (6.25) |  |
| High | 13 | (28.89) | 7 | (43.75) |  |
| Intermediate | 31 | (68.89) | 6 | (37.5) |  |
| Low | 1 | (2.22) | 1 | (6.25) |  |
| unprecised | 0 | (0.00) | 1 | (6.25) |  |
| ***Disease status at transplantation for AML (n=33/14)*** |  |  |  |  |  |
| *CR1* | *22* | *(66.67)* | *8* | *(61.54)* |  |
| *CR2* | *7* | *(21.21)* | *3* | *(23.07)* |  |
| *Not in CR* | *4* | *(12.12)* | *2* | *(15.38)* |  |
| ***Cytogenetic prognosis for AML at diagnosis (n=33/14)***  ***(according to ELN 2017)*** |  |  |  |  |  |
| *Good* | *5* | *(15.15)* | *1* | *(7.70)* |  |
| *Intermediate* | *18* | *(54.54)* | *6* | *(46.15)* |  |
| *Poor* | *10* | *(30.30)* | *5* | *(38.46)* |  |
| *Unknown* | *0* | *(0.00)* | *1* | *(7.70)* |  |
| ***Disease status at transplantation for MDS (n=12/3)***  ***(according to IWG 2006)*** |  |  |  |  |  |
| *CR* | *5* | *(11.36)* | *3* | *(17.64)* | ***P=0.15*** |
| *PR* | *4* | *(9.09)* | *0* | *(0.00)* | ***P=0.28*** |
| *Stable disease* | *3* | *(6.81)* | *0* | *(0.00)* | ***P=0.06*** |
| *Progressive disease* | *0* | *(0.00)* | *0* | *(0.00)* | ***p=0.33*** |
| ***Cytogenetic prognosis for MDS ( IPSS-R) (n=12/3)*** |  |  |  |  |  |
| *Very good* | *0* | *(0.00)* | *0* | *(0.00)* | ***P=0.12*** |
| *Good* | *7* | *(58.33)* | *0* | *(0.00)* |  |
| *Intermediate* | *1* | *(8.33)* | *0* | *(0.00)* | ***P=0.33*** |
| *Poor* | *1* | *(8.33)* | *0* | *(0.00)* | ***P=0.55*** |
| *Very poor* | *3* | *(25.0)* | *3* | *(100)* | ***P=0.33*** |
| **Conditioning regimen** |  |  |  |  | P=0.28 |
| **MAC** | 4 | (8.9) | 3 | (18.75) |  |
| **RTC/ RIC** | 41 | (91.1) | 13 | (81.25) |  |
| **Type of donor** |  |  |  |  | P=0.82 |
| MRD | 10 | (22.22) | 4 | (25.00) |  |
| MUD | 35 | (77.78) | 12 | (75.00) |  |
| **Median donor age, years (range)** | 31 | (18-75) | 45 | (19-70) | P=0.09 |
| **ABO incompatibility** |  |  |  |  | P=0.59 |
| None | 23 | (51.11) | 11 | (68.75) |  |
| Minor | 11 | (24.44) | 3 | (18.75) |  |
| Major | 8 | (17.78) | 1 | (6.25) |  |
| Bidirectional | 3 | (6.67) | 1 | (6.25) |  |
| **CMV risk** |  |  |  |  | P= 0.47 |
| High risk (donor negative to recipient positive) | 15 | (33.33) | 8 | (50.00) |  |
| Low risk (donor negative to recipient negative) | 13 | (28.89) | 3 | (18.75) |  |
| Intermediate risk (donor positive) | 17 | (37.78) | 5 | (31.25) |  |

Abbreviations: AML, acute myeloid leukemia; CMV: cytomegalovirus; CR: complete remission; ELN, European Leukemia Network; IPSS-R: revised international prognostic scoring system; IWG: international Working Group; MAC: myeloablative conditioning; MDS: myelodysplastic syndrome; MRD: matched related donor; MUD: matched unrelated donor; PR: partial remission; RIC: reduced intensity conditioning; WHO World Health Organization.

Table 2. Patient outcomes.

| **Outcomes** |  | NR (n=45) | |  |  | R (n=16) | |  | p-value |
| --- | --- | --- | --- | --- | --- | --- | --- | --- | --- |
| **Engraftment (n, %)** |  | 44 | (97.78) |  |  | 14 | (87.5) |  | P=0.10 |
| **Median chimerism (% bone marrow recipient cells) at D+30** |  | 1 | (0-10) |  |  | 1.5 | (0-40) |  | P=0.92 |
| **Median chimerism (% bone marrow recipient cells) at D+100** |  | 1 | (0-8) |  |  | 14 | (0-79) |  | **P<0.0001** |
|  |  |  |  |  |  |  |  |  |  |
| **Acute graft-versus-host disease (n, %)** |  |  |  |  |  |  |  |  | P=0.34 |
| Stages 0-1 |  | 31 | (68.89) |  |  | 13 | (81.25) |  |  |
| Stages 2-4 |  | 14 | (31.11) |  |  | 3 | (18.75) |  |  |
| **Chronic graft-versus-host disease (n, %)** |  |  |  |  |  |  |  |  | P=0.57 |
| mild |  | 9 | (20.00) |  |  | 1 | (6.25) |  |  |
| moderate |  | 4 | (8.89) |  |  | 1 | (6.25) |  |  |
| severe |  | 5 | (11.11) |  |  | 0 | (0.00) |  |  |
| **Infections requiring treatments after engraftment** |  |  |  |  |  |  |  |  | P=0.39 |
| EBV requiring rituximab treatment |  | 10 | (22.22) |  |  | 8 | (50.00) |  |  |
| CMV requiring antiviral treatment |  | 15 | (33.33) |  |  | 2 | (12.5) |  |  |
| Aspergillosis/mucormycosis |  | 2 | (4.44) |  |  | 0 | (0.00) |  |  |
| Cerebral abscess |  | 1 | (2.22) |  |  | 0 | (0.00) |  |  |
| Cellulitis |  | 1 | (2.22) |  |  | 0 | (0.00) |  |  |
| Catheter-related bloodstream infection |  | 1 | (2.22) |  |  | 0 | (0.00) |  |  |
| Sepsis shock |  | 1 | (2.22) |  |  | 0 | (0.00) |  |  |
| Covid 19 |  | 3 | (6.66) |  |  | 1 | (6.25) |  |  |
| **Main causes of death (n, %)** |  |  |  |  |  |  |  |  | P=0.002 |
| Severe sinusoidal obstructive syndrom |  | 1 | (2.22) |  |  | 0 | (0.00) |  |  |
| Poor graft function |  | 1 | (2.22) |  |  | 0 | (0.00) |  |  |
| Acute GVHD |  | 3 (1 with cerebral abscess) | (6.66) |  |  | 0 | (0.00) |  |  |
| Chronic GVHD |  | 2 (1 with COVID 19) | (6.66) |  |  | 0 | (0.00) |  |  |
| Relapse |  | 2 | (6.66) |  |  | 15 | (93.75) |  |  |

**
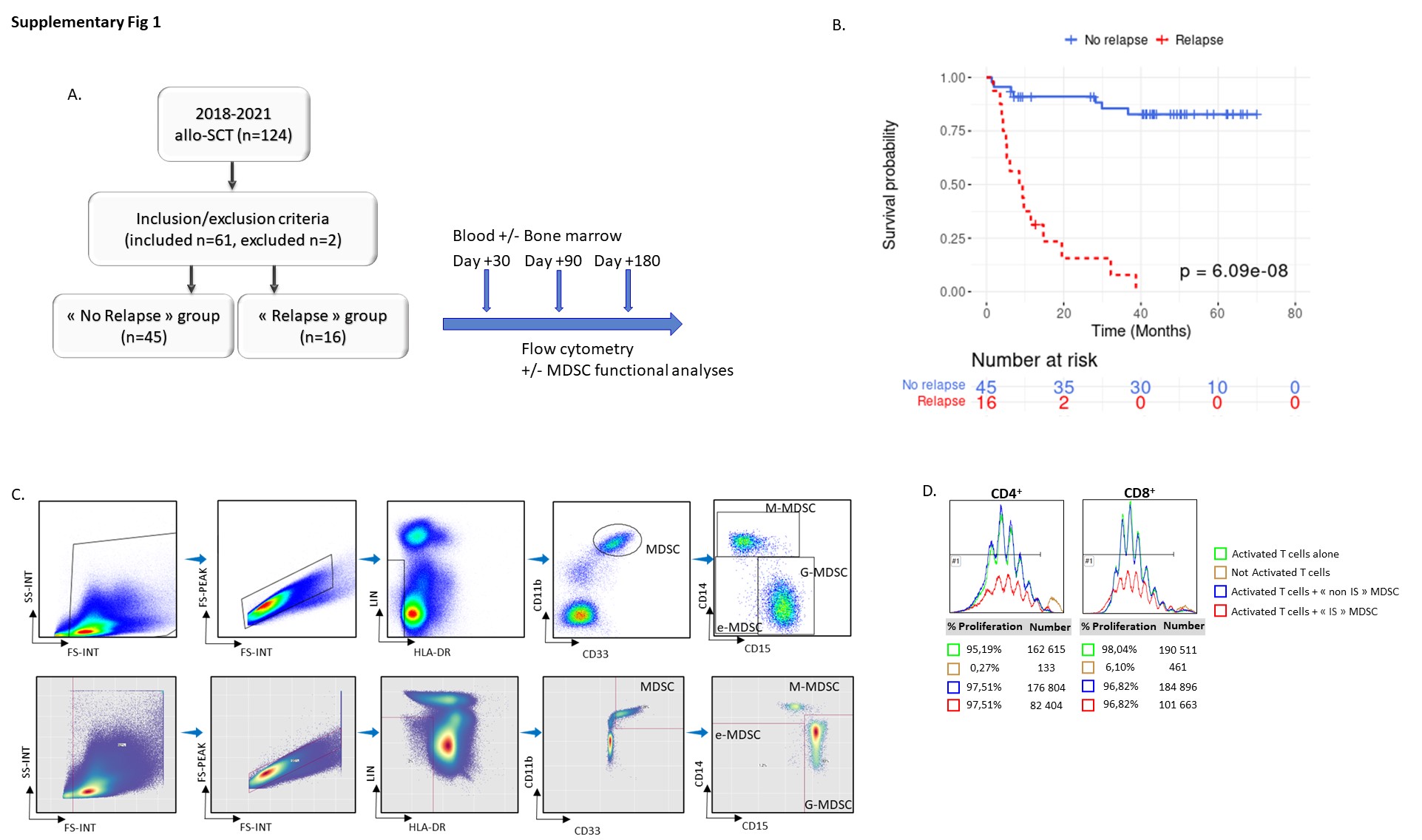
Supplementary figure**
